# Impact of Environmental Factors on the Regulation of Cyanotoxin Production

**DOI:** 10.3390/toxins6071951

**Published:** 2014-06-25

**Authors:** Thangavelu Boopathi, Jang-Seu Ki

**Affiliations:** Department of Life Science, Sangmyung University, Seoul 110-743, Korea; E-Mail: iamboopathy@gmail.com

**Keywords:** cyanotoxin, microcystins, nodularin, cylindrospermopsin, anatoxins and saxitoxins

## Abstract

Cyanobacteria are capable of thriving in almost all environments. Recent changes in climatic conditions due to increased human activities favor the occurrence and severity of harmful cyanobacterial bloom all over the world. Knowledge of the regulation of cyanotoxins by the various environmental factors is essential for effective management of toxic cyanobacterial bloom. In recent years, progress in the field of molecular mechanisms involved in cyanotoxin production has paved the way for assessing the role of various factors on the cyanotoxin production. In this review, we present an overview of the influence of various environmental factors on the production of major group of cyanotoxins, including microcystins, nodularin, cylindrospermopsin, anatoxins and saxitoxins.

## 1. Introduction

Cyanobacteria are a group of oxygenic photoautotrophic Gram negative bacteria which are morphologically diverse and ubiquitous in nature [1,2]. They are also known as one of the primitive oxygen-producing organisms on Earth with the fossil records of ~3.5 billion years [3]. Cyanobacteria capable of fixing atmospheric nitrogen, solubilize phosphorous and sequester iron which gives them greater adaptability to diverse terrestrial and aquatic environments with various nutrient levels [4]. The occurrence of cyanobacterial bloom in fresh water sources is considered as an environmental problem worldwide. In particular, toxic cyanobacterial blooms in freshwater systems around the world have increased in frequency and severity [1,5]. However, nutrient enrichment of both freshwater and marine systems by anthropogenic activities promoted the growth of cyanobacteria, and increased the incidence of harmful algal blooms [1,6,7,8]. 

Cyanotoxins are diverse group of secondary metabolites produced by various genera of cyanobacteria which are toxic to most of the eukaryotic organisms including algae, plants, cattle and humans (Table 1) [4,9]. Many bloom-forming cyanobacteria are known to produce various toxins which have drastic impact on the ecosystem and its functions [9,10]. In addition, cyanobacterial blooms create a threat to drinking and recreational water by producing harmful toxins and stinking compounds [11]. In addition, the bloom formation can cause hypoxia and disrupt food webs in the ecosystem [11]. 

Cyanotoxins are broadly classified based on their modes of action in invertebrates such as hepatotoxins, neurotoxin, and dermatotoxins (aplysiatoxin, lyngbyatoxin-a). This review mainly emphases on the regulation of major cyanobacterial hepatotoxins and neurotoxins. Cyanobacterial hepatotoxins include microcystin (MCs), nodularin (NODs) and cylindrospermopsin (CYN). However, the latter one has both cytotoxic and neurotoxic potentials [9,12,13]. In addition, anatoxins (ATXs) and saxitoxins (STXs) are neurotoxins which are produced by various cyanobacterial species [14]. Based on their chemical composition, cyanotoxins can be grouped in to cyclic peptides (MCs and NODs), alkaloids (ATXs, STXs, CYN, aplysiatoxin, lyngbiatoxin-a) and lipopolysaccharides [13,15]. Most of the cyanotoxins are not extracellular, withf exception of CYN, but when cyanobacterial cells lyses, these toxins can be released at high concentrations into the environment [12]. Further, various cyanotoxins can also exhibit allelopathic properties that may have adverse effects on surrounding communities include other bacteria and phytoplankton [16,17].

The current scenario in global climate change and increased CO_2_ concentrations can greatly influence the microbial communities in various environments, particularly in aquatic ecosystems [10,18]. The cyanobacterial community may also be altered by various physical/chemical factors, including change in environmental conditions such as climate change and it is essential to understand and predict the responses of these communities [7,19]. After comparing 42 years of incidence data of cyanobacteria and other eukaryotic phytoplankton from the small subalpine Castle Lake in northern California, Park *et al.* [20] found that the cyanobacterial biovolume was increased with increasing water temperatures. However, it has been shown that the diatoms and other phytoplankton groups did not reveal any significant change with summer water temperatures [20]. In a survey of 143 lakes along a latitudinal transect ranging from subarctic Europe to southern South America with varying trophic states revealed the significant increase in cyanobacterial biomass with increasing temperature whereas total phytoplanktonic biomass remain unchanged [21]. However, the factors involved in the increase of toxin production and increased occurrence of toxin-producing cyanobacterial species are still unclear. When polar cyanobacterial mat samples were grown at increased temperatures favored the growth of toxin producing genera, even at nutrient limiting conditions [18]. Globally, the trend of increased growth of toxin-producing *Microcystis* spp. has been observed in the recent years [1,22]. 

**Table 1:** Nature of cyanobacterial toxins (see text for further details [15,23,24,25]).

Toxins	Variants	Toxin producing cyanobacterial genera	Toxic mechanism	Health effects
Microcystin	Over 85 variants	*Anabaena*, *Anabaenopsis*, *Aphanizomenon*, *Merismopedia*, *Microcystis*, *Oscillatoria*, *Phormidium*, *Synechococcus* and *Planktothrix*	Hepatotoxic, inhibits eukaryotic protein phosphatases	Gastrointestinal, liver inflammation, and hemorrhage and liver failure leading to death, pneumonia, dermatitis
Nodularin	8 variants	*Nodularia* and *Nostoc*	Hepatotoxic, inhibits eukaryotic protein phosphatases	Gastrointestinal, liver inflammation, and hemorrhage and liver failure leading to death, pneumonia, dermatitis
Cylindrospermopsin	3 variants, Cylindrospermopsin 7-epicylindrospermopsin 7-deoxycylindrospermopsin	*Cylindrospermopsis*, *Anabaena*, *Aphanizomenon*, *Oscillatoria*, *Raphidiopsis*, *Umezakia* and *Sphaerospermopsis*	Hepatotoxic, cytotoxic, neurotoxic; inhibition of glutathione synthesis, protein synthesis and cytochrome P450	Gastrointestinal, liver inflammation and hemorrhage, pneumonia, dermatitis
Anatoxin-a	3 variants, Anatoxin-a, homoanatoxin-a Anatoxin-a(s)	*Anabaena*, *Aphanizomenon* and *Oscillatoria*	Neurotoxic, mimics the neurotransmitter acetylcholine	Tingling, burning, numbness, drowsiness, incoherent speech, respiratory paralysis leading to death
Saxitoxin	20 variants	*Anabaena Aphanizomenon*, *Cylindrospermopsis*, *Lyngbya*, *Planktothrix*, *Raphidiopsis* and *Scytonema*	Neurotoxic, blocks voltage-gated Na^+^ channels	Tingling, burning, numbness, drowsiness, incoherent speech, respiratory paralysis leading to death

Moreover, toxin producing cyanobacteria not only withstand various environmental stresses but also produce elevated levels of toxins under stressful conditions [26,27]. Cyanobacteria blooms generally comprised of various species including toxin and non-toxin producers as well as nitrogen and non-nitrogen-fixers [27]. The environmental factors such as temperature, light intensity, pH, nutrients, salinity, ultraviolet radiation, wind, trace metals and environmental pollutants can influence the growth of the cyanobacterial species and their cyanotoxin production [14]. Therefore, understanding the influence of environmental changes on cyanobacterial bloom formation and toxin production is crucial for the management of toxic cyanobacterial bloom. In this review, we summarize the effect of regulatory molecules and influence of various environmental factors on the biosynthesis of five major cyanotoxin groups. Although the studies pertaining to the influence of environmental factors on the regulation of cyanotoxin production at molecular level are scarce, this review attempts to summarize the available reports. 

## 2. Microcystins (MCs) & Nodularins (NODs)

The hepatotoxins MCs and NODs are found to be similar with regard to structure and mode of action, and are hence discussed together in this section. These toxins were found to target liver and brain [28,29] and inhibit the protein phosphatase [23] that will result in the accumulation of phosphorylated proteins in the liver which triggers cell death through necrosis and hemorrhage [15,30]. Although the toxicity was considered based on the mixture of toxic variants of the above toxins, MC-LR (leucine and arginine variant) and NOD-R (arginine variant) are being referred as standards and the lethal dose 50 (LD50) of MC-LR is 25 to 150 µg/kg and LD50 for NOD-R is 50 to 150 µg/kg [23]. The mechanism behind the toxicity of MC-LR was studied recently in detail by [31]. Their study indicated the induction of protein kinase and phosphorylation of proteins associated with microfilaments in human liver cell line HL7702 by MC-LR [31]. MCs were also reported as having tumor inducing property [23]. Recently, MCs were isolated from *Planktothrix rubescens* Strain No80 which shown to inhibit protein phosphatases at nano molar range [32]. While MCs are reported in varied regions like Asia, Europe, North Africa, North America and Scandinavian countries, NODs appear to be confined to Australia, New Zealand and the Baltic Sea [23,33,34]; since MCs have been produced by so many species of cyanobacteria (*Microcystis*, *Oscillatoria*, *Aphanizomenon*, *Merismopedia*, *Nostoc*, *Anabaena*, *Phormidium*, *Planktothrix* and *Anabaenopsis*) and production of NODs were reported only with *Nodularia spumigena*, *Nodularia sphaerocarpa* and also with *Nostoc* species recently [13,14,15,35,36,37].

### 2.1. Synthesis and Regulation

**Figure 1:**
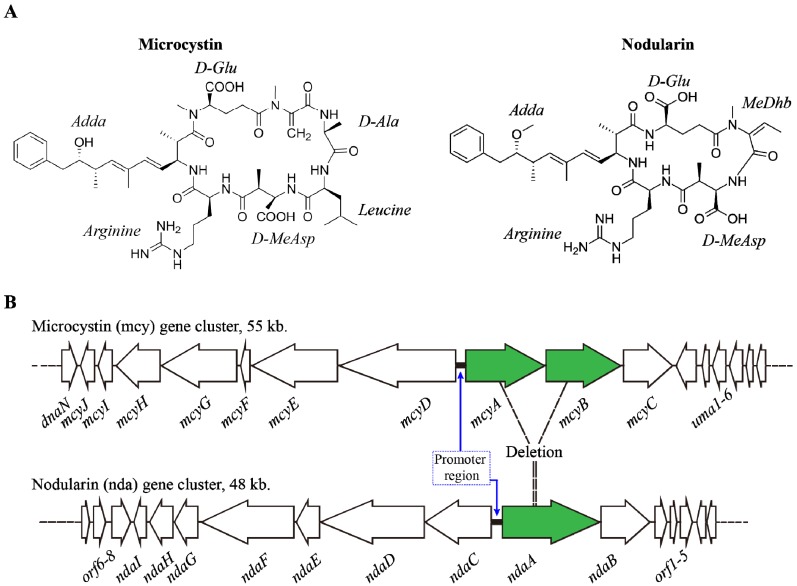
(**A**) Shows the structure of Microcystin-LR and Nodularin; (**B**) Showing the gene cluster *mcy* encoding Microcystin and *nda* cluster which encodes Nodularin; possible derivation of *nda* from *mcy* region is also given. Promoter regions were highlighted with bold lines in both *mcy* and *nda* cluster. See text for further details [38].

MCs and NODs are cyclic heptapeptides and pentapeptides which possess the unusual Adda amino acid (3-amino-9-methoxy- 2, 6, 8-trimethyl-10-phenyl-4, 6-dienoic acid) with diene conjugate respectively (Figure 1A). They are produced non-ribosomally from *mcy* and *nda* gene clusters respectively (Figure 1B). To date, more than 85 variants of MCs were identified from various cyanobacterial species [14,33]. Biosynthesis of MCs, NOD and other cyanobacterial toxins and their toxic nature was reviewed by Pearson *et al.* [15].

MCs are synthesized by enzyme complexes including non-ribosomal peptide synthetase (NRPS) and polyketide synthase (PKS) [14]. In *M. aeruginosa* PCC7806, *mcy* (*mcyABCDEFGHIJ*) gene cluster (55 kb) contains two polycistronic operons with 10 genes enclosing *mcyABC* and *mcyDEFGHIJ* and a bidirectional promoter has been observed between *mcyA* and *mcyD* (Figure 1B) [13]. Detailed study has been done on *mcy* regulation [12,15,39]. *mcy* is regulated by a complex network system including limitations of nutrients, dual transcriptional regulator and factors inducing stress (light, *etc.*). These factors are involved in the regulation of MCs synthesis. A study by Wood *et al.* [40] reported that significant changes were observed with *mcyE* transcript levels in a *Microcystis* bloom. Further, their study suggested the influence of environmental factors in toxin production [40]. Organization of the ORFs of the *mcy* cluster is not the same between the following organisms: *Microcystis*, *Anabaena* and *Planktothrix* [14]. As hypothesized by Rouhiainen *et al.* [41], *mcy* of *Microcystis* and *Planktothrix* might be derived from *mcy* of *Anabaena* along with some gene reorganization events. In addition, Christiansen *et al.* suggested that [42] *mcy* cluster could be derived from *nda* cluster by some gene additions. However, some genes of the toxin gene cluster might be conserved throughout the evolution.

### 2.2. Environmental Factors Regulating MCs Synthesis

Biosynthetic gene cluster of microcystin synthetase *mcy* has been shown with binding sites for the following transcriptional regulators namely, NtcA (nitrogen responsive regulatory protein), FurA (a ferric uptake regulator) and RcaA (redox-active photoreceptor protein) [39,43,44,45]. NtcA is a dual transcriptional regulator; based on the binding site it may up/down regulate the candidate genes. The consensus sequence for NtcA binding is 5' GTA N8 TAC 3' with highly conserved bases at the end ~22 bp is found at the upstream of the −10 region [46,47]. This recognition sequence is surrounded by A/T bp and the dual regulation of NtcA is based on the number of the intervening spacer nucleotides [48]. New binding sites for NtcA concerning the promoter of *mcy* were also studied in detail [49]. Nitrogen limitation was shown to increase the production of MC by enhancing the transcription of *mcy*. As reviewed by Ermilova and Forchhammer, [50] under N-limitation, cellular levels of 2-oxoglutarate (2-OG) increased and PipX (P_II_-interacting protein X) was released from the signal transduction protein P_II_ and readily available to bind with NtcA and 2-OG to form the complex. This complex helps in the binding of NtcA towards its target sequences to regulate gene expression. However, with enough N, a complex formed between PipX and P_II_ which cannot activate NtcA [50]. Ginn *et al.* [44] also confirmed that the expression of *ntcA* was more (~4 fold) under N-limitation than with excess N in *M. aeruginosa* PCC7806 which is a microcystin producer and in the same study induction of *mcyB* was also reported under N-limitation. Kuniyoshi *et al.* [49] demonstrated the binding affinity between NtcA and promoter of *mcy* cluster *(mcyDA*, *mcyE* and *mcyH*) by electrophoretic mobility shift assay (EMSA); they also confirmed that this binding affinity between NtcA and *mcyA* promoter was further promoted 2.5 fold with increased 2-OG at 25 μmol photons m^−2^·s^−1^. It was confirmed that the 2-OG also acts as the signaling molecule to sense the balance between C and N and thus regulates MC production [51]. Negative regulation of NtcA was also suggested on *mcyABC* promoter, since one of the consensus sequences of NtcA showed similarity with the binding site of *gor* repressor in *Anabaena* PCC7120 [52].

Reports regarding relations between nitrate concentrations and MC production have provided contradictory results as described below, and this may be due to methanol extraction in which protein-bound toxin is not being measured. Although we mentioned above that N-limitation was found to have positive impact on MC production [53], in the unicellular, non-nitrogen-fixing cyanobacterial species, more MC production was reported with increased nitrate concentration [54,55,56]. In *M. aeruginosa* PCC7806, elevated nitrate concentrations could only increase the growth rate and did not change MC production [57]. Tonk *et al.* [58] studied the effect of N availability on MC production; when they added Leucin (N poor amino acid), microcystin LR synthesis increased and microcystin-RR increased when they added the arginine (N rich amino acid) in *Planktothrix agardhii* strain 126/3 [58]. It was also shown that CO_2_ and nitrogen enrichment was the main reason for increased production of N-rich microcystin-RR variants in *Microcystis* blooms [59]. Proteomics studies of toxic and nontoxic *M. aeruginosa* strains revealed that genes involved in carbon dioxide-concentrating mechanism (CCM) (*CcmK3* and *CcmL*), genes involved in nitrogen uptake and metabolism (*P_II_* and *NrtA*) and the redox balance regulating protein coding genes (*NdhK* and *TrxM*) showed differential expression pattern and they seem to play a vital role in maintaining C:N in the cell which can modulate MC production [39]. These studies also indicated the effect of MCs in the proteome of *M. aeruginosa*. MCs producing cyanobacterial strains were found to have more adaptation ability under unfavorable conditions than the mutant non-toxin producers [60,61]. Recently, induction of MC production in response to increased nitrogen in *M. aeruginosa* has been reported [62]. Community structure of cyanobacteria which consists of MC-RR producing *M. aeruginosa*, MC-LA producing *M. wesenbergii* and MC-YR producing *Aphanizomenon flos-aquae* was influenced by environmental factors and especially forms of N-source, and this will determine the toxic nature of the bloom [63].

Research by Ding *et al.* [64] strongly suggested that UVB radiation supports the growth of MC producing cyanobacterial strains. They also indicated that non-MC producers are vulnerable to radiation when compared with MC producers [64]. Researchers showed that light also plays an essential role in MC production; differences in light intensity could change the transcription start sight of *mcyA* [43,65] and this was not observed with various nitrogen concentrations. Sevilla *et al.* [57] suggested that light was found to have more impact on MC production than nitrogen. In P. agardhii, increased light intensity altered the ratio between MC variants; production of microcystin-DeLR was greater while the production of microcystin-DeRR decreased with more light ~60 μmol m^−2^·s^−1^, among them microcystin-DeLR was found to be more toxic than microcystin-DeRR [66]. Initial induction in transcription of *mcyD* was observed under more light intensity [30,67] µmol photons m^−2^·s^−1^ [68] and 30 µmol photons m^−2^·s^−1^ [65] and reduction was also reported. High light intensity shows an advantage in growth rate and toxin production in microcystin-producing *M. aeruginosa* PCC7806 and their work also confirmed that MCs protect the cells against photo oxidation and changes in the nutrition did not show any changes in MC production under high light [69]. Although the relationship between oxidative stress and MC is not clearly understood, induction of *mcy* transcription, binding of MC with RbcL and sensitivity of *mcy* mutants revealed that MC might have a protective role against high light intensity and oxidative stress conditions [26]. Prevention of chlorosis was observed with MC synthesis under light stress and production of pigments (chlorophyll a, b-carotene, zeaxanthin and echinenone) was under greater light stress in wild type of *Microcystis aeruginosa* when compared with mutant MC non producer cells. MC was found to protect the cells by binding with the large subunit of RuBisCo (*rbcL*), when oxidative stress induced by high light intensity [12]. Reports discussed above strongly suggested the vital role of NtcA in the regulation of *mcy* and *rbcL*, carbon cycling and in oxidative stress. The relation between photosynthesis and MC production was studied in detail [68,70]; it shows that cells need active photosynthesis to produce more toxins. 

There are discrepancies in studies associated with FurA and MC production, although the binding site of FurA has been reported in *mcy* promoter. As per Alexova *et al.* [39,45], reduction in transcription of *mcyA* was seen while MCs production was induced due to iron starvation, although FurA usually negatively regulates the target genes in the presence of ferrous iron. Increased MC production and *mcyD* transcription was observed under iron limitation [71]; but a study by Utkilen and Gjølme, [54] indicated that more MCs were produced with more available iron. These contradictions may be due to the methodology they used in their study. By considering all the factors mentioned above, growth rate of cyanobacterial strains might influence the production of MCs and thus environmental factors might play a vital role in the production of these toxins.

### 2.3. Environmental Regulation of Nodularin

Regarding NOD synthesis and regulation, NOD was also synthesized by NRPS and PKS enzymes. *nda* cluster consists of nine ORFs (*ndaA-ndaI)* with a bidirectional regulatory promoter; *ndaAB*, ORF1, ORF2 and *ndaC* has been transcribed in two polycistronic mRNAs (Figure 1B) [38]. As reviewed by Pearson *et al.* [15], ORF1, ORF2 and ORF3 were found to be present at the downstream of *ndaAB* genes which are associated with *nda* cluster by encoding a putative transposase, putative high light-inducible chlorophyll-binding protein and putative heat shock repressor protein respectively; they also suggested that ORF2 might be associated with NOD synthesis under light stress and ORF3 might play a role in transcriptional regulation of *nda* cluster under heat stress. The *nda* cluster is predicted to be derived from the region between *mcyA-A2* and *mcyB-C2* domain by deletion (Figure 1B) [34,72]. To date, eight variants of NOD have been reported [73].

*Nodularia spumigena* blooms were reported predominately under higher phosphorus, moderate salt concentrations and low N:P ratios [73]. Recent reports concluded that NOD production is being controlled by three major factors including biological nitrogen fixation, light stress and phosphate limitation (see below). Occurrence of the binding site of NtcA (nitrogen responsive regulatory protein) in the upstream region of *nda* in *N. spumigena* NSOR10 and *N. spumigena* CCY9414, and also in the heterocyst forming genes of *Anabaena* PCC7120 indicated the role of nitrogen fixation in nodularin synthesis [74]; *hetR* gene from *Nodularia* and *Nostoc* species were found to be regulated by NtcA [34]. Jonasson *et al.* [75] reported that *nda* cluster was inducible with phosphate starvation and down-regulated by ammonia supplementation, but it did not change intracellular/extracellular concentrations of nodularin; ammonia supplementation also down-regulates the transcription of *ndaF* and *nifH* genes; reduction in nitrogen fixation was reported in *N. spumigena* with ammonia [75,76]. These results confirmed the essentiality of nitrogen fixation in NOD production. However, it purely depends on environmental factors, because more production of NOD was observed in aquatic nitrogen fixing species, *N. spumigena*, but very low production of NOD has been reported from terrestrial *Nostoc* spp. [72]. Differences in carbon and nitrogen fixation rates might be the cause for this change in NOD production as reported by [77]. Light stress does play a vital role in NOD synthesis, ORF2 (co-transcribed with *nda* cluster) which codes for high light inducible chlorophyll-binding protein (HLIP) is associated with light stress in *N. spumigena* NSOR10 and *N. spumigena* CCY9414 [38,78]. NOD synthesis was enhanced by elevated temperature (25–28 °C), more light (45–155 µmol photons m^−2^·s^−1^) and high phosphate concentration (200–5500 μg·L^−1^) while high salinity and high inorganic nitrogen concentrations were found to inhibit NOD production [79]. All these studies also suggested that combination of environmental factors plays a major role in NOD production.

## 3. Cylindrospermopsin

### 3.1. Characteristics

Cylindrospermopsin (CYN) is an alkaloid toxin containing guanidiono and sulfate groups produced by several filamentous cyanobacteria including *Cylindrospermopsis raciborskii*, *Anabaena bergii*, *Aphanizomenon ovalisporum*, *Aph. flos-aquae*, *Oscillatoria* sp. PCC6506, *Raphidiopsis curvata*, *Sphaerospermopsis aphanizomenoides* and *Umezakia natans* [12,13,80,81,82,83,84]. Among CYN producing cyanobacteria, *C. raciborskii* is the well-known CYN producing cyanobacteria which contains both toxin producing and non-toxin producing strains [80,85,86]. However, in recent years, CYN has also been reported from temperate countries, such as Germany and France and also from boreal environments [87,88]. It is also shown that CYNs have been accumulated in the environment over time [89]. CYN production is similar to the synthesis of MCs, where toxin synthesis is regulated by genes coding for polyketide synthase and peptide synthetase [80]. 

### 3.2. Structure and Toxicity

**Figure 2:**
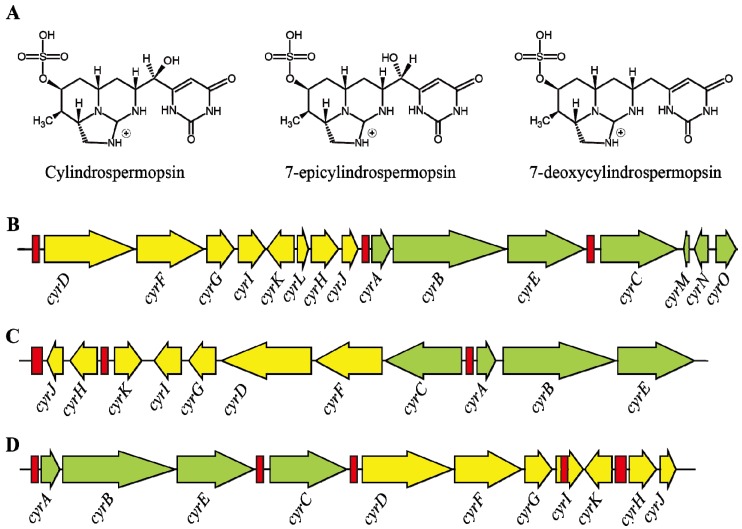
(**A**) Showing the structure of cylindrospermopsin and its variants; (**B**–**D**) showing the gene cluster of *cyr* which encodes cylindrospermopsin from various syanobacterial species namely; (**B**) *C. raciborskii* AWT205; (**C**) *Aphanizomenon* sp. strain 10E6 and (**D**) *R. curvata* CHAB1150, respectively; red bars indicates the transposase coding region or vestiges thereof [96].

CYN, is an alkaloid (415 Da) containing a tricyclic guanidine unit and a uracil moiety which is mainly involved in toxicity [90]. In addition, two other variants of this toxin have also been identified; 7-epicylindrospermopsin which differs only by the orientation of the hydroxyl group close to the uracil moiety [91], and deoxycylindrospermopsin, which is characterized by a missing oxygen atom related to the initial hydroxyl group close to uracil moiety (Figure 2A) [92]. However, deoxycylindrospermopsin appears to be non-toxic [93]. The zwitterionic structure of CYN makes it a highly polar compound and thus highly water-soluble [94]. Chiswell *et al.* [94] reported that CYN has poor stability in crude algal extract in which 90% of the toxin has been degraded within 3 days when exposed to sunlight. In contrast, CYN showed more than 10 days of half-life in high purity water which indicates that pH and temperature may play a major role in the degradation of toxin [94]. In contrast, Wormer *et al.* [95] reported that the CYN was not degraded by the naturally occurring microbial communities even after 40 days and this may lead to accumulation of CYN in water bodies and increase the risk.

CYN has hepatotoxic, nephrotoxic and general cytotoxic properties and it also acts as a potential carcinogen [97,98,99]. CYN primarily inhibit protein, cytochrome P450 and glutathione synthesis which may lead to cell death [97,98,99,100,101]. CYN have affected mainly the liver, kidney, lung and intestine in the mouse bioassays [102]. When compared to MCs, CYN causes cell death through irreversible inhibition of protein synthesis [99]. The other effects of CYN are micronucleus induction, chromosome loss, tumor initiation and fetal toxicity [98,103,104]. However, various human and animal intoxications have been reported including famous Palm Island mystery disease [14,15,105,106]. In the Palm Island of Australia, during 1979, 139 children and 10 adults suffering from hepato-enteritis were admitted to the hospital after the consumption of drinking water treated with algicide to eliminate cyanobacteria; this outbreak was caused by the release of CYN [106].

### 3.3. Transcription of Cylindrospermopsin Synthetase Gene

The cylindrospermopsin biosynthesis has been carried out by the *cyr* gene cluster which was studied in several cyanobacteria including *C. raciborskii* strains, *Aphanizmoenon* sp. 10E6 and *Oscillatoria* sp. PCC 6506 (Figure 2B) [81,107,108]. The size of the *cyr* cluster spans around 42 kb which contains 15ORFs (*cyr A-O*) which encode all the necessary functions for biosynthesis, regulation and export of CYN [108]. A recent study comparing genomes of both toxic and non-toxic strains revealed that the *cyr* cluster is completely absent in non-CYN producing strains; however, mutations and alterations in *cyr* cluster did not affect the toxin synthesis [85]. This cluster is highly conserved among various CYN producing cyanobacterial genera. However, the gene arrangements in the cluster vary between different genera [12]. The *cyrO* of *cyr* cluster has been absent in *Oscillatoria* PCC 6506; however, it contains sequences for two additional transposases and an ATP-grasp type protein which has no apparent role in CYN biosynthesis [81]. It also suggested that the horizontal gene transfer of *cyr* cluster is possible because of the following properties, including possession of high GC contents, difference in flanking genes, highly conserved sequences across genera and the presence of transposases [107]. CYN biosynthesis involved many complex enzymes including NRPS/PKS-type enzymes, amidino transferase and other tailoring enzymes [109]. Even though CYN biosynthesis gene cluster has been characterized in several cyanobacteria, only a handful of reports are available on studies focusing on its promoter structure and transcriptional organization. Further, within this *cyr* cluster, sequence motifs for the binding of global nitrogen regulator NtcA were also found. In *C. raciborskii* AWT205, both sides of the *cyr* cluster are flanked by *hyp* genes, which are regulated by NtcA and involved in maturation of hydrogenases [14,67]. It appears that *cyr* cluster could have been inserted in to the *hyp* genes, as these *hyp* genes generally present as clusters in cyanobacteria. Further, the presence of transposase-like sequences within the *cyr* cluster supports this view [14]. It was also suggested that the regulation of *cyr* cluster by NtcA was a consequence of insertion of whole *cyr* cluster in to *hyp* genes [108]; however, this view is yet to be supported by experimental evidences. Two transcription start sites each for the genes *aoaA* (homologue of *cyrA*) and *aoaC* (homologue of *cyrC*) were found in *Aph. ovalisporum* and their functions under different growth conditions such as nitrogen availability and light intensity were studied. However, from these results it was suggested that the transcription of *aoa* genes may be regulated by two promoters; one constitutive promoter and another for differential regulation under different environmental conditions [110]. It is also found that the synthesis of CYN and deoxy-CYN were a constitutive process and both the concentration and ratio between the release of CYN and deoxy-CYN varied among different strains of *C. raciborskii* [111]. In addition, proposed transcript initiation sites of *aoaA* and *aoaC* genes were specifically bound by a protein-like transcriptional regulator AbrB, which suggests the involvement of such protein in the regulation of CYN production and the genomes of *Oscillatoria* sp. PCC6506 and *C. raciborskii* CS-505 showed the sequences for AbrB-like proteins [81]. Regulator binding sites with similar conserved sequences were reported in the upstream region of *aoaC*/*cyrC* genes and this study also supported that the mechanism of transcriptional regulation might be same in these two organisms.

### 3.4. Role of Environmental Factors in CYN Production

Production of CYN was being influenced by nutrients and majorly by light. N source (NO_3_^−^ NH_4_^+^, N_2_) has a major impact on CYN synthesis and discrepancies were also reported regarding CYN production and N-source since the studies were done with different growth conditions. An investigation about CYN and N source was started by Saker and Neilan [112] in *C. raciborskii*. In study by Shalev-Malul *et al.* [110], up regulation of *aoaA* (~2.5-fold) and *aoaC* (4-fold) was reported while there was no change in the remaining CYN concentration and there no significant changes (except slight down regulation under ammonia) were observed in the transcription of *cyr* genes (*cyrB*, *cyrI*, *cyrJ* and *cyrK*) under various N sources in *C. raciborskii* CS-505 [113]. When the cultures of *C. raciborskii* cells were grown with lack of a fixed-nitrogen source, CYN was increased more while the growth rate was low; CYN was less with NH_4_^+^ source while the growth rate was high; more over significant changes were not observed with NO_3_^−^ source [112]. Phosphate limitation also plays an important role in CYN production but contradictions were reported regarding phosphate limitation and CYN production. Bacsi *et al.* [114] indicated the decrease in CYN under phosphate limitation and the increased expression of alkaline phosphatase secretion in *Aph. ovalisporum*. However, in study [115], an increase in CYN production was observed with phosphate limitation in *Aph. ovalisporum.* The authors also found induced level of alkaline phosphatase secretion and Pi-uptake transporters when they used spent *Aph. ovalisporum* medium and this was not the case with purified CYN alone. However, a phosphate addition both alone and with NO_3_ has increased the CYN concentrations in mesocosm experiments dominated by *C. raciborskii* [116]. Further, the proportion between *cyrA* and *16S* genes in the *C. raciborskii* population was also found to be higher in P addition treatments with higher intracellular CYN concentrations, which suggests that phosphate addition may favor toxic strains than non-toxic strains [116]. However, a shift between toxic and non-toxic strain dominance has already been reported. Changes in biovolume of *C. raciborskii* were lower than changes in intracellular toxin concentration suggesting a shift in the proportion of toxic and non-toxic cells. The importance of shifts in strain dominance of *C. raciborskii* has previously been proposed after comparing intracellular CYNs, the *cyrC* toxin gene and the *rpoC1* gene of *C. raciborskii* in a bloom population [117]. In addition, intracellular CYN concentrations were found to be higher at increased P concentration in a Saudi lake [118]. However, the molecular mechanisms lying behind the alteration in strain differences in *C. raciborskii* to the addition of P addition are unclear and necessitate more research. Regarding the light intensity, high light intensity (85 µmol photons m^−2^·s^−1^) was found to affect *cyr* transcription initially (after 8 h) and increased the same after given time (24 and 48 h) in *Aph. ovalisporum* [110]. The combination of high light intensity (140 µmol photons m^−2^·s^−1^) and medium lack of nitrogen increased the production of CYN while the lower growth rate was indicated under high light intensity [119]. Intracellular and extracellular measurement of CYN in *Oscillatoria* sp. PCC 6506 under different light conditions showed that total CYN content is highest during the exponential growth phase at intermediate light level (10 µmol photons m^−2^·s^−1^) and also during the stationary growth phase at extreme lower and higher light levels with the extracellular form ranged between 56% and 96% of the total CYN concentrations [120]. CYN synthesis was reported to be less with sulfate exposure in *Aph. ovalisporum* [114]. Further, *cyrJ* was up-regulated by the addition of an allelopathic compound pyrogallic acid at the concentration of 4 mg·L^−1^ in *C. raciborskii* [121]. These studies clearly indicate that various environmental factors influence CYN production by various cyanobacteria. However, indepth analysis in the future is required to understand how these environmental variables alter CYN production and strain composition.

## 4. Anatoxins (ATXs)

### 4.1. Structure and Occurrence of ATXs

**Figure 3:**
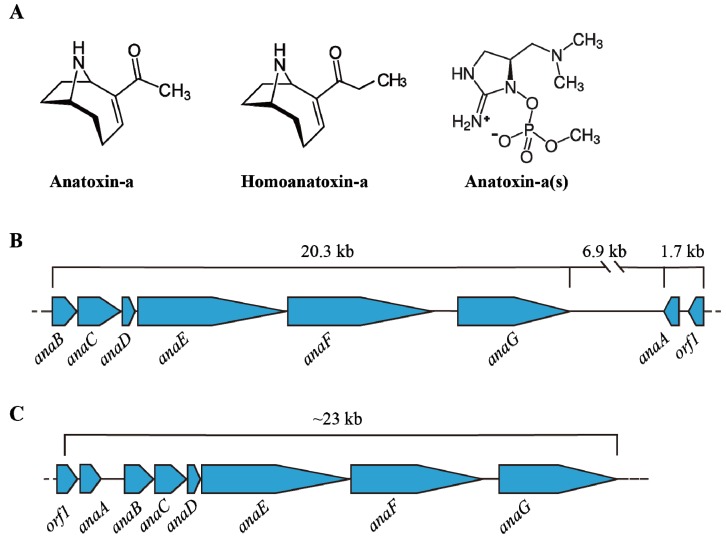
(**A**) Shows the variants of Anatoxins; The *ana* cluster encodes anatoxin from (**B**) *Anabaena* sp. strain 37 and (**C**) *Oscillatoria* sp. PCC6506 (adapted from [134]).

Anatoxins are alkaloids produced by various cyanobacteria including, *Anabaena*, *Oscillatoria* and *Aphanizomenon* [122,123]. To date, three common anatoxins have been identified from cyanobacteria namely anatoxin-a (ATX-a), homoanatoxin-a (hATX-a) and anatoxin-a(s) (ATX-a(s)). Although ATX-a (165 Da) and hATX-a are secondary amines, hATX-a varies from ATX-a by its methylation at the ketone structure; ATX-a(s) (252 Da) has different structure which is a phosphate ester of a cyclic *N*-hydroxyguanidine (Figure 3A) [24,123]. ATX-a is soluble in water and not stable under alkaline condition and sunlight; ATX-a was also not stable under high UV-B radiation and high temperature; stability of ATX-a(s) was also changed with alkaline condition and heat [124,125,126]. These neurotoxins all inhibit the activity of acetylcholinesterase and affect muscle function by causing constriction, convulsion and respiratory dysfunction, which eventually leads to death [127]. Different animal poisonings were reported with ATX-a and its variants [128,129,130]; hypersalivation was reported with ATX-a(s) [131]. Although no guidelines were reported with ATX toxicity in water, the animal experiments showed LD50 for ATX-a is 375 µg/kg (via i.p. route in mice) and >5000 µg/kg via oral [132]. ATX-a(s) was more effective than ATX-a; LD50 of ATX-a(s) is 20 µg/kg [127]. The study [133] has given the detection protocol for ATX-a and its three analogues based on fluorescent polarization (FP) method.

ATX-a(s) has been produced only by *Anabaena* sp. while ATX-a has been produced by various genera of cyanobacteria including *Anabaena*, *Aphanizomenon*, *Planktothrix*, *Microcystis*, *Oscillatoria* and *Cylindrospermum.* ATX-a has been reported widely including in America, Asia, Africa and in Europe; however, the presence of ATX-a(s) was observed only from a few regions of the United States, Scotland, Denmark and Brazil [132].

### 4.2. Transcription of Ana Cluster

ATX-a and its variants were synthesized by the addition of prolin by NRPS (non-ribosomal peptide synthetases) and subsequent chain extension and cyclization was done by PKS (polyketide synthases) [122]. ATX gene cluster *anaA-anaG* and ORF1 was described by [134] (Figure 3B). It was reported from *Oscillatoria* sp. PCC6506 [135] and *Anabaena* sp. 37 [134] with high sequence similarity and different organization in these spp. The study [134] described the *ana* gene cluster in *Anabaena* sp. 37; *ana* cluster contains four or five operons with characteristic sequence motifs in the upstream without any RNA Polymerase binding site. They also indicated that two clusters of *ana* gene (29 kb) have been described in the opposite direction with 6 kb spacer region. Molecular methods have been developed to detect anatoxins [134]. To date, studies on the regulation of *ana* cluster is in its infancy and further studies are needed.

### 4.3. Environmental Impact on ATX

Variation in synthesis of ATXs was reported with nutrient limitation, light, temperature and different growth phase [65,136,137,138,139]. These toxins were not related to cell growth, while MC is associated with growth rate as mentioned by [53]. N starvation could increase ATX production as we already mentioned above for other cyanobacterial toxins [14]. Reduction of ATX-a synthesis was reported in *Anabaena* and *Aphanizomenon* strains with high temperatures despite of their growth rate [138]. Studies [138,140] revealed that sub optimal level of light and temperatures were helped to produce more ATXs and also lower light was associated with extracellular ATX production in *Anabaena.* The work in [141] suggested the role of temperature and media composition in ATX production; synthesis of ATX-a was observed in *Ocillatoria* sp. PCC6506 in BG11 media at 22 °C without CO_2_ enrichment while the synthesis of hATX-a was observed in BG1110 media supplemented with 10 mM NaHCO_3_ at 25 °C with CO_2_ enrichment in the same. Addition of green algal extract (*Chlamydomonas reinhardtii*) enhanced the toxin level in *A. flos-aquae* batch cultures as reported by [142]. 

## 5. Saxitoxin

### 5.1. Structure and Toxicity of STXs

**Figure 4:**
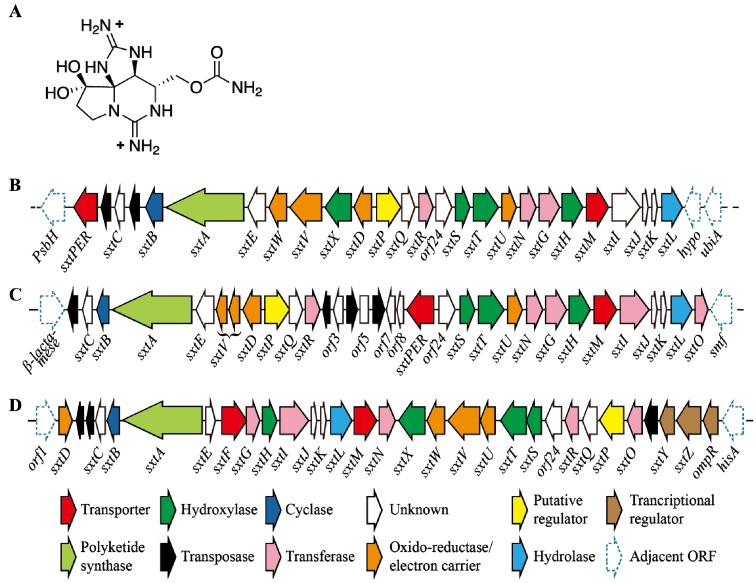
(**A**) Shows the structure of saxitoxin; (**B**–**D**) showing the gene cluster encodes saxitoxin from *Aphanizomenon.* sp. NH-5, *Anabaena circinalis* AWQC131C and *Cylindrospermopsis raciborskii* T3 respectively (not drawn to scale) [154].

Saxitoxin (STXs) comes under the class of alkaloids which share tricyclic backbone with different side group moieties. Fifty seven analogues of saxitoxin have been reported [143]; they have been also known as paralytic shellfish toxins (Figure 4A). Toxicity of analogues of saxitoxin mainly depends upon the interactions between guanidinium ions and the target molecules [144]. These neuro toxins are known to inhibit the function of voltage-gated channels in vertebrates [145,146]. These toxins have been produced by various cyanobacterial genera including *Anabaena*, *Aphanizomenon*, *Cylindrospermopsis*, *Lyngbya*, *Planktothrix*, *Raphidiopsis*, *Scytonema* and dinoflagellates [14].

STXs come under a family of tricyclic compounds (241 to 491 Da); about 20 variants of STXs were reported including singly sulphated or doubly sulphated, non-sulphated and decarbamoyl derivative STXs [132,147]. Their stability in water is about 90 days [148] and further degrading into high toxic variants under high temperature determined their toxicity. These neurotoxins are the main cause for the paralytic shellfish poisonings and they showed LD50 of 10 µg/kg (in mice) as reported by [23]. They were found to inhibit the sodium ion channels and affect nerve function; which causes severe paralyses with respiratory failure and finally leads to death [24]. Mortality events (109 cases) by paralytic shellfish poisonings have been reported in North and Central America [23], although no reports are available about human intoxication through the water. Although the chlorination was found to reduce the acute toxicity most of the cyanotoxins, including MC, CYN, NOD, and STX, the actual reaction and toxic nature of by-products was only identified in MC and CYN and not identified in case of STX and NOD with chlorine [132]. 

### 5.2. Regulation of STX Production

To date, very few investigations have been done regarding saxitoxin regulation. Bioinformatic analyses of Kellmann *et al.* [149] indicated that the synthesis of STX begun with the multifunctional polyketide biosynthesis enzyme (*SxtA*). Only a few studies are evident about STX genes; to date STX biosynthetic gene cluster has been reported from five genera of cyanobacteria [107,108,149,150,151] and 33 genes involved in STX biosynthesis, transport and regulation were also reported in their study. The size of these gene clusters was 25.7 kb (*Raphidiopsis brookii* D9) to 36 kb (*Lyngbya wollei*). However, the toxic profile of the each strain is determined by the position/presence or absence of genes in the above cluster (Figure 4B–D). Studies on *C. raciborskii* T3, revealed the presence of *sxtY*, *sxtZ* and *ompR* genes which are being involved in STX regulation. More experimentation is required to know the actual mechanism behind this, but these proteins showed homology with the phosphate stress-activated two-component signal transduction system [149]. Further understanding about STX warrants new experiments regarding its genomics and proteomics, but methodology for 2D-PAGE have been developed in *C. raciborskii* and *Raphidiopsis* sp. [152]. Recently, genetically predicted biosynthetic intermediate of STX such as Int-A, Int-C2 and D2 have been identified and quantified in *Anabaena circinalis* [153], which support the biosynthetic route completely as proposed by Kellmann *et al.* [149].

### 5.3. Impact of Environmental Factors

Synthesis of STX has been changed by the environmental factors; nutrients, light, growth temperature and salt concentrations found to have a potential impact on STX production (Table 2). The high ratio of nitrogen to phosphorous influenced the STX production in *C. raciborskii*; high N:P treatment has increased the STX production nearly one fold when compared to low N:P [155]. However, with increased concentrations of nitrogen, a lower level of STX has been observed in *Raphidiopsis brookii* [156]. Production of STX was recorded as more with high light intensity (100 µmol photons m^−2^·s^−1^) when compared with 50 and 150 µmol photons m^−2^·s^−1^ light in *C. raciborskii* T3; reduction of STX in dark was also reported in *C. raciborskii* T3. Moreover, these studies suggested the role of circadian clock in STX production [157,158]. Contradictory results were reported regarding the growth temperature and STX production as we mentioned in other toxins; when compared with optimal growth temperature of 25 °C, STX production was being more (especially the extracellular toxin concentration) at 19 °C, although the growth rate was less in *C. raciborskii* strain C10 [159]. However, a high temperature (28 °C) seems to induce the STX production in *Aphanizomenon* sp. LMECYA 31 more than the usual growth temperature 22 °C [160]. Work of Pomati *et al.* [161] revealed that extracellular NaCl has increased the production of STX and this was also confirmed by the presence of saxitoxin-producing cyanobacteria *C. raciborskii* T3 in waters with high conductivity; their study also suggested that this STX production might have impact on sodium channels in the cells [161]. STXs necessitate more research since we have only a poor understanding about STX gene regulation and pathways to date.

**Table 2:** Effect of various factors on the regulation of various cyanotoxins (see text for further details).

Toxin	Gene cluster	Up regulating factors	Down regulating factors
MCs	*mcy*	active photosynthesis	FurA
		*N*-limitation	
		more nitrate	
		NtcA	
		FurA	
		RcaA	
		2-OG	
		high light intensity	
NODs	*nda*	Nitrogen fixation	Ammonia supplementation high salinity high inorganic nitrogen
		NtcA	
		Phosphate starvation	
		light stress	
		high temperature	
CYNs	*cyr*/*aoa*	lack on fixed N source	Ammonia as N-source high light intensity (initially) phosphate limitation
		phosphate limitation	
		high light intensity	
		(more incubation)	
ATXs	*ana*	*N*-starvation	High temperature
		sub optimal light	
		sub optimal temperature	
		green algal extract	
		(*Chlamydomonas reinhardtii*)	
STXs	*stx*	High light intensity	High Nitrogen Dark conditions
		High temperature	
		sub optimal temperature	
		Extracellular salt (NaCl)	

## 6. Conclusions and Future Remarks

Recently, expanding cyanobacterial blooms has been a major target of researchers because of their harmful toxin production. These toxins are regulated by so many factors including nutrients’ limitations (Nitrogen, phosphorous and Carbon), light, heat, oxidants and other components. Studies regarding cyanobacterial toxins and the role of environmental factors in these toxins should be compiled to understand clearly about the effect of toxins in the environment. As we stated above, the ability of toxin producers to adapt to all environmental conditions was very high when compared with non-toxin producers; these toxins were also found to play a vital role in their growth rate. In this review we have compiled the essential information regarding the synthesis and regulation of cyanobacterial toxins to date (see Table 2). We stress in this review that stressors are found to have an important role in the regulation of these toxins. Although the cyanobacterial research started a few decades ago, studies regarding the gene regulation and the impact of environmental factors in toxin production are still in their infancy because of the complexity in the system. Moreover, studies describing about toxin synthesis and regulation were not done under similar conditions and thus could not be compared to many of these studies. Although the laboratory experiments provide essential information regarding toxins, more field experiments are needed to know the role of environmental factors in them, although this is usually associated with many practical concerns. In the future, further molecular studies are needed to provide a clear view about the factors influencing toxin production.
